# Survival and Independent Predictors of Mortality Following Coronary Artery Bypass Graft Surgery in a Single-Unit Practice in the United Kingdom Over 20 Years

**DOI:** 10.7759/cureus.38413

**Published:** 2023-05-01

**Authors:** Aziz Momin, Redoy Ranjan, Oswaldo Valencia, Adam Jacques, Pitt Lim, David Fluck, Tuan P Chua, Venkatachalam Chandrasekaran

**Affiliations:** 1 Cardiac Surgery, St. George's University Hospitals NHS Foundation Trust, London, GBR; 2 Cardiac Surgery, Bangabandhu Sheikh Mujib Medical University, Dhaka, BGD; 3 Cardiology, Ashford and St Peter’s Hospitals NHS Foundation Trust, Chertsey, GBR; 4 Cardiology, St. George's University Hospitals NHS Foundation Trust, London, GBR; 5 Cardiology, Royal Surrey NHS Foundation Trust, London, GBR

**Keywords:** survival benefits, mortality, multiple arterial graft, long term outcome, total arterial cabg, bilateral internal mammary artery, coronary artery bypass graft

## Abstract

Background: The types of graft conduits and surgical techniques may impact the long-term outcomes of patients after coronary artery bypass graft (CABG) revascularization. This study observed a long-term survival rate following CABG surgery over 20 years in the United Kingdom.

Methods: A total of 2979 isolated CABG patients were studied from 1999 to 2020, and postoperative data were obtained from the hospital-recorded mortality by the data quality team of the information department. Postdischarge survival was estimated using the Kaplan-Meier method, and statistical significance was obtained with log-rank tests and the Gehan-Breslow test, and the Holm-Sidak method was used for multiple pairwise comparisons.

Results: The study observed male predominance (80%), and the median age was statistically significant (P <0.001) among the groups, 66 years (interquartile range 58-73) and 72 years (interquartile range 66-78) in survivor and non-survivor groups, respectively. In the Holm-Sidak method analysis, the best survival rate (mean 18.7 years) was observed in the total arterial group with significantly decreased survival for the mixed arterial and venous group (mean 16.12 years) and only the vein group (10.44 years). The Cox regression model observed that the New York Heart Association (NYHA) class III-IV (HR 1.57), chest re-exploration (HR 2.14), preoperative dialysis (HR 3.13), and redo surgery (HR 3.04) were potential predictors of the postoperative mortality (P ≤0.05).

Conclusion: In our series over 20 years, albeit off-pump and on-pump CABG observed similar survival rates, the total arterial myocardial revascularization population has significantly better long-term survival benefits.

## Introduction

Coronary artery bypass graft (CABG) surgery is the most commonly performed myocardial revascularization procedure for multivessel coronary artery disease [1,2]. However, published papers observed that the types of graft conduits are directly related to graft patency rate, potentially influencing the postoperative outcome, especially the long-term survival rate [2,3]. Recently published research articles and meta-analyses demonstrated that total arterial myocardial revascularization (TAR) provides better longevity and improves postoperative outcomes, especially early graft failure, health-related quality of life, and redo-CABG surgery [3-6].

The use of the left internal mammary artery (LIMA) has an excellent long-term graft patency rate; however, the bilateral internal mammary artery (BIMA) graft in CABG is still under evaluation, especially regarding long-term graft patency and survival benefits [4-9]. However, a recently published meta-analysis demonstrated better survival benefits and long-term outcomes for multiarterial CABG surgery [10]. Only a few randomized controlled trials (RCTs) evaluated early postoperative outcomes that were limited to small samples or survival, and most of the extensive observational studies have only described in-hospital outcomes following TAR-CABG surgery [11-15].

This study aimed to determine the potential hazard factors and long-term survival benefits of TAR over 20 years in an extensively large, population-based cohort in the United Kingdom.

## Materials and methods

This study retrospectively evaluated a total of 2979 consecutive patients who underwent isolated CABG at St. George's University Hospital NHS foundation trust from April 1999 to March 2020, and the last census was on May 2021 in a single surgeon team practice. Preoperative, intraoperative, and postoperative data were collected prospectively and validated. Dates of death were obtained from the hospital-recorded mortality. Completeness of mortality and validation of NHS numbers was carried out by the data quality team of the information department. In accordance with the National Research Ethics Service, this retrospective study was completed as a Service Evaluation and Improvement, using data already collated as patients received their usual care and did not require research ethics committee approval. Study inclusion criteria were isolated CABG with or without prior history of heart surgery, and patients with concomitant valvular, congenital heart diseases were excluded from the study. Each patient appears only once in the series, and this study population was divided into two study groups: survivor and non-survivor population, which were further subdivided into two sub-groups: Subgroup-I: 2190-patients were off-pump CABG and Subgroup-II: 789-patients were on-pump CABG.

The primary endpoint was to analyze the long-term survival rate following isolated CABG surgery, and the secondary outcome was to establish the hazard factors associated with poor survival and postoperative morbidity. Total arterial revascularization techniques utilized single or bilateral internal mammary artery with radial artery, and complete myocardial revascularization indicates that all significant coronary lesions were revascularized with desired distal anastomosis, either an artery or venous grafts. The mean follow-up duration was 60±8.5 months, with outpatient department visits or phone calls, especially during the covid pandemic.

Surgical technique and patient selection criteria 

All elective CABG patients aged ≤65 years (in the UK, those with age >65 years are considered older persons) were evaluated for total arterial CABG, and the LIMA was the choice of conduit for LAD lesions. However, emergency cases and the absence of suitable arterial conduits (calcified radial artery, irreparable conduits injury by trainee) required the use of venous conduits. Furthermore, to ensure good conduit quality, we preoperatively assessed the radial artery with Allen's test, which was cross validated while harvesting the radial artery. Moreover, we ensured to harvest internal mammary artery (IMA) in a skeletonized fashion and to its terminal branches (superior epigastric artery and musculophrenic artery) to avoid short mammary conduits. Our usual revascularization technique was to perform a LIMA-RIMA "Y" graft fusing 8/0 prolene for left-sided distal anastomosis and radial artery for right-sided grafts to PDA and RCA. Although this study is based on a single-unit practice of two surgeons, off-pump CABG was performed by an experienced UK consultant with over 20 years of experience as a consultant, while the on-pump CABG surgeon had almost 10 years in the field as a UK consultant. A quality control assessment was carried out to validate the off-pump and on-pump CABG samples.

Statistical analysis

IBM SPSS Statistics for Windows, Version 25 (Released 2017; IBM Corp., Armonk, New York, United States) was utilized to analyze the data, and data were analyzed for the survivors versus the deceased population. Univariate analyses of dichotomous, categorical, and continuous data were performed to determine similarities or differences between patients who died or survived over time. Tabulations of dichotomous and categorical variables were compared using the Chi-square test or Fisher's exact test, as appropriate, and the hazard ratio was also calculated to observe the likelihood of postoperative mortality. The distribution of continuous variables was assessed for normality with the Shapiro-Wilk test, and Mann-Whitney U tests were used to compare both groups (medians and IQR). Several models of multivariate regression analyses were made with all independent categorical and continuous variables that appeared significant for postoperative mortality. A Kaplan-Meier survival analysis was performed with clinically relevant groups, and hazard ratios were calculated using the Cox regression proportional hazards model to identify the predictor of death. Significance was obtained with the Gehan-Breslow test, and the Holm-Sidak method was utilized for multiple pairwise comparisons. A P-value ≤0.05 is considered statistically significant. 

## Results

The study evaluated a total of 2979 consecutive ischemic heart disease patients (80% male) undergoing isolated CABG surgery, and the median age was statistically significant (P <0.001) among the groups, 66 years (IQR 58-73) and 72 years (IQR 66-78) in survivor and non-survivor groups, respectively (Table 1). Risk factor evaluation observed a significant difference between study groups, specifically NYHA Class III & IV, previous myocardial infarction (MI), diabetes, hypertension, smoking, chronic pulmonary disease, Stroke/TIA, extracardiac arteriopathy, preoperative AF, cardiogenic shock, preoperative dialysis, IABP, and previous heart surgery except for CABG between survivor and non-survivor groups (P<0.05). Furthermore, LV dysfunction (low EF), preoperative creatinine, and EuroSCORE II were significantly higher in the non-survivor group (P<0.05). In quality control analysis between the off-pump versus on-pump CABG technique, we observed no significant difference in the EuroSCORE II and mean graft number.

**Table 1:** Sociodemographic variables of the study population (N=2979) IQR- Interquartile range; NYHA Class- New York Heart Association (NYHA) Functional Classification, PCI- percutaneous coronary intervention, COPD- chronic obstructive pulmonary disease, TIA- transient ischemic attack, PVD- peripheral vascular disease, AF- atrial fibrillation, IABP- intra-aortic balloon pump, LVEF- left ventricular ejection fraction. P value reached from the Chi-square Test except the P value^a^, which is reached from the Mann-Whitney U Test. P value ≤0.05 is considered statistically significant.

Variables		Survivor ( N= 2306)	Non Survivor (N= 673)	P value
Age (Median; IQR)		66 (58-73)	72 (66-78)	<0.001^a^
Sex	Male	81.0%	80.5%	0.8
	Female	19.0%	19.5%	
Risk Factors	NYHA Class III-IV	8.6%	18.0%	<0.001
	Recent MI	48.3%	56.5%	<0.001
	Previous PCI	19.1%	17.4%	0.31
	Diabetes on Insulin	25.9%	31.9%	0.002
	Uncontrolled Hypertension	69.3%	76.1%	<0.001
	Smoking	59.1%	64.0%	0.02
	COPD	5.8%	12.3%	<0.001
	Stroke/TIA	7.2%	12.2%	<0.001
	PVD	10.0%	22.6%	<0.001
	Preoperative AF	4.6%	10.5%	<0.001
	Left Main Stem Disease	27.4%	30.8%	0.08
	Cardiogenic Shock	0.3%	1.6%	<0.001
	Preoperative Dialysis	0.6%	2.5%	<0.001
	Preoperative Ventilated	0.1%	0.7%	<0.001
	Preoperative IABP support	2.8%	6.5%	<0.001
	Previous heart operation except CABG	2.7%	4.9%	<0.001
	Previous CABG	0.04%	0.3%	0.28
Nature of Surgery	Elective	63.9%	59.3%	<0.001
	Urgent	33.1%	34.9%	
	Emergency	3.0%	5.8%	
Left Ventricular Function	LVEF > 50%	78.9%	67.5%	<0.001
	LVEF 30-50%	18.6%	25.8%	
	LVEF < 30%	2.5%	6.7%	
Body Mass Index (Median; IQR)		27 (25-30)	27 (24-29)	<0.001^a^
Pre-op creatinine (Median; IQR)		84 (73-96)	96 (80-118)	<0.001^a^
EuroSCORE II (Median; IQR)		1.38 (1.05-2.05)	2.85 (2.02-2.55)	<0.001^a^

**Figure 1:**
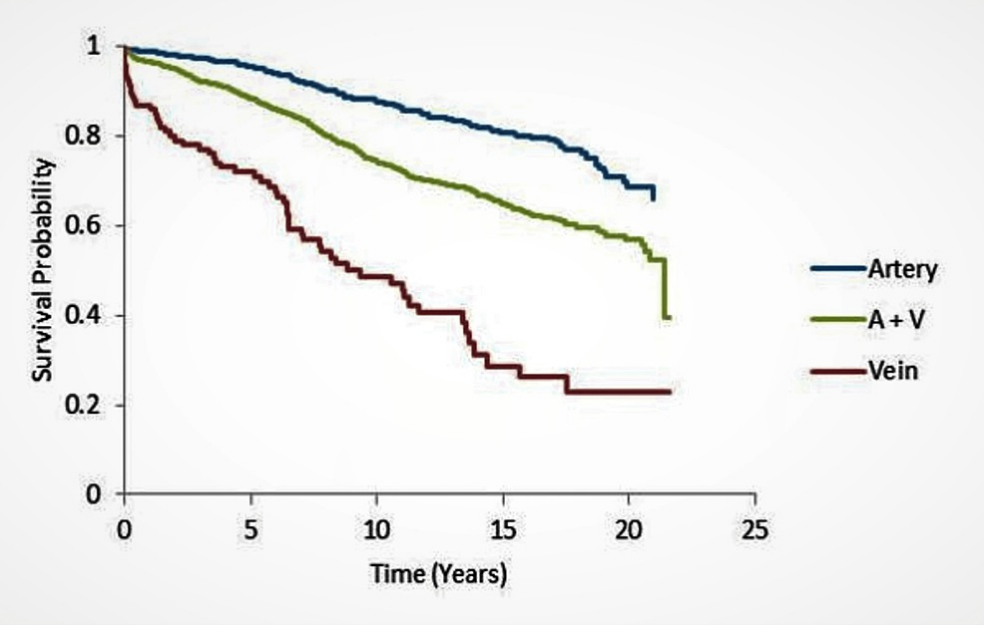
Kaplan-Meier survival curve based on the type of conduits

We found the mean survival time 18.70 (95% CI 18.26-19.14), 16.12 (95% CI 15.71-16.53), and 10.44 (95% CI 8.78-12.10) years among total arterial, mixed arterio-venous, and only vein groups, respectively (Figure 1).

**Figure 2:**
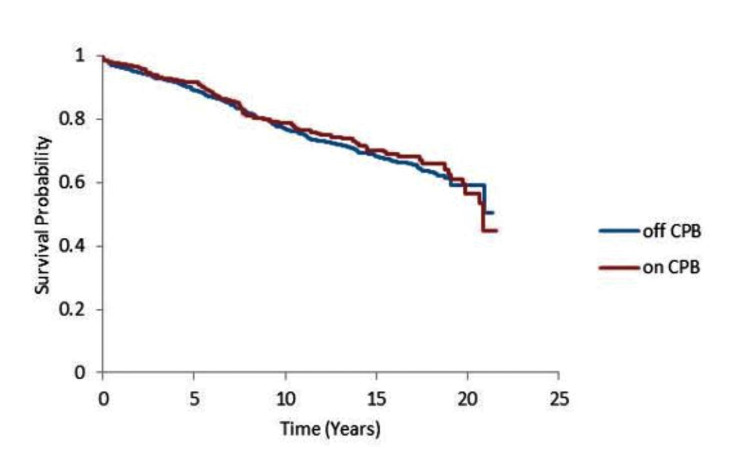
Kaplan-Meier survival curve for the operative technique

As shown in Figure 2, Kaplan-Meier curve analyses the long-term survival benefits according to the surgical techniques, and the mean survival rate following OPCABG and ONCABG was 16.7 and 17.1 years, respectively, which is statistically insignificant (P=0.43).

According to Table 2, Cox regression hazards model observed age (HR 1.08), uncontrolled hypertension (HR 1.31), multi-vessel CAD (HR 1.43), recent MI (HR 1.54), NYHA class III-IV (HR 1.57), peripheral vascular disease (HR 1.61), preoperative dialysis (HR 3.13), chest re-exploration (HR 2.14), and redo surgery (HR 3.04) were potential predictors of the mortality of the study population (P ≤0.05).

**Table 2:** Cox regression model predicting independent predictors of mortality over 20 years HTN- Hypertension, COPD- chronic obstructive pulmonary disease, CAD- coronary artery disease, MI- myocardial infarction. P value ≤0.05 is considered as statistically significant.

Variables	Hazards ratio	95% Confidence interval (CI)		P value
		Lower CI	Upper CI	
Age at Operation	1.08	1.07	1.09	<0.001
Uncontrolled HTN	1.31	1.10	1.57	0.003
COPD	1.43	1.13	1.80	0.003
Multi-vessel CAD	1.43	1.22	1.69	<0.001
Recent MI	1.54	1.22	1.93	<0.001
NYHA Class III-IV	1.57	1.29	1.92	<0.001
Peripheral vascular Disease	1.61	1.34	1.93	<0.001
Chest re-exploration	2.14	1.52	3.03	<0.001
Preoperative Dialysis	3.13	2.38	4.13	<0.001
Redo surgery	3.04	2.13	4.35	<0.001

## Discussion

The current study evaluated the largest sample of 2979 consecutive CABG patients and observed that total arterial CABG surgery provides a significantly higher (18.7 years) long-term survival rate than the venous group. Nevertheless, despite the technique, either off-pump or on-pump CABG, chest re-exploration, redo surgery, and preoperative dialysis are the most significant independent predictors of mortality following CABG surgery.

In 1986, Loop et al. first observed the survival benefit of IMA graft in CABG surgery, and to date, the standard utilized CABG conduits are the IMA, radial artery, and greater saphenous vein [2]. However, the preferences of CABG conduits are crucial to the long-term outcome because the patency of a conduit is closely related to the nature of the atherosclerotic disease, types of conduits, and an uneventful postoperative course [3-5]. In several published papers, researchers have demonstrated the superiority of bilateral internal mammary artery over single internal mammary artery (BIMA versus SIMA) graft on long-term survival and major adverse cardiac-related events [5-8]. In a study, Tatoulis and co-workers observed that long-term survival benefits are directly related to the graft patency rate, and BIMA graft has ≥90% patency rates in contrast to venous grafts, of which 75% are re-stenosed by 10 years of follow-up which is similar to this current study results [9].

In the absence of large RCTs, several nonrandomized trials and small follow-up studies observed that the survival benefit of total arterial bypass grafting appears to continue through the second decade of postoperative follow-up [7-10]. Although Taggart and coauthors found no statistically significant difference in survival or event-free survival at ten years of follow-up among single versus bilateral IMA grafts in the arterial revascularization trial (ART), Professor Taggart proposed complete coronary revascularization and a second arterial graft, precisely, a radial artery graft to the second most crucial lesion following LAD artery as integral to long-term survival benefits, a concordance to the current study results [4]. In a recent study, Gaudino et al. [11] and Rocha et al. [12] have also found superior survival benefits when the radial artery is utilized as a second CABG conduit compared to the saphenous vein.

In a recent meta-analysis by Rayol and colleagues evaluating a total of 22,746 patients [13], the pooled HR for long-term survivability over 10 years was higher in the TAR group (HR 0.676, 95% CI 0.59-0.78), similar to existing small published data [5-9,14,15]. In a recent study, Kurlansky et al. observed better long-term outcomes and quality of life as measured by the SF-36 in patients undergoing CABG using bilateral internal mammary artery conduits [16]. The actuarial survival at 15 years was ~54% and ~51% for women and men, respectively, comparable to this study's results. Moreover, Tatoulis et al. [9] observed that the long-term graft patency of the right IMA (RIMA) graft is excellent, about 90% over 10 years, which is equivalent to the left IMA for identical territories and remains free of atheroma, supported by other authors [8,12,15-19]. 

Furthermore, Lytle and colleagues [20] reported that the long-term survival advantage of BIMA grafting continued to diverge out to 20 years following CABG surgery, and they found a significantly better (P<0.001) survival rate of 89%, 67%, and 50% at 7, 15, and 20 years, respectively BIMA versus SIMA grafts, similar to other study results [21-23]. In a recent study, Davierwala et al. [24] and Locker et al. [25] found that multiple arterial graft's more desirable configuration provides lower long-term all-cause mortality following CABG after a median follow-up of 12 and 15 years, respectively, which also supported the current study findings.

The main strength of the current study is the analysis of the largest CABG sample over a long (20 years) period in a well-organized system, the National Health Service, United Kingdom, with minimum loss of patient follow-up. However, like any ambispective observational study, this study has a few limitations, including a need for standardized outcomes definitions, analysis of both on-pump and off-pump CABG cases, and the absence of angiographic graft patency data. However, being the largest prospectively collected datasets, a real-life registry and experiences over 20 years mitigate the limitations with available clinical follow-up data. Moreover, there was no heterogeneity in the association between surgical techniques and postoperative outcomes, and the study results remained robust in the sensitivity analyses. Insofar as both off-pump and on-pump CABG surgeons are experienced in the field and working independently for more than 10 years, which mitigates the potential outcome bias. We recommended using arterial conduits utilizing internal mammary arteries and radial artery grafts over venous conduits, especially for those aged <65 years.

## Conclusions

Despite complete myocardial revascularization, the types of conduits utilized in CABG surgery significantly impact long-term survival benefits, especially with total arterial CABG using bilateral internal mammary arteries and radial artery grafts having an excellent long-term survival rate. We found that the total arterial CABG population greatly benefitted from multiple arterial grafts and a survival rate of ~19 years, significantly higher than the only venous graft group, where mean survival was only 10.44 years. We also observed that redo surgery, pre-existing renal failure requiring dialysis, and chest re-exploration were the most significant predictors for long-term mortality following CABG surgery.
